# Supplementary material for: MAGI1 Mediates eNOS Activation and NO Production in Endothelial Cells in Response to Fluid Shear Stress
Source: Cells. 2019 Apr 27;8(5):388. doi: 10.3390/cells8050388 (PMC6562810; doi:10.3390/cells8050388)
Supplement: Supplementary file 1 [file cells-08-00388-s001.pdf]

Supplementary figure legends

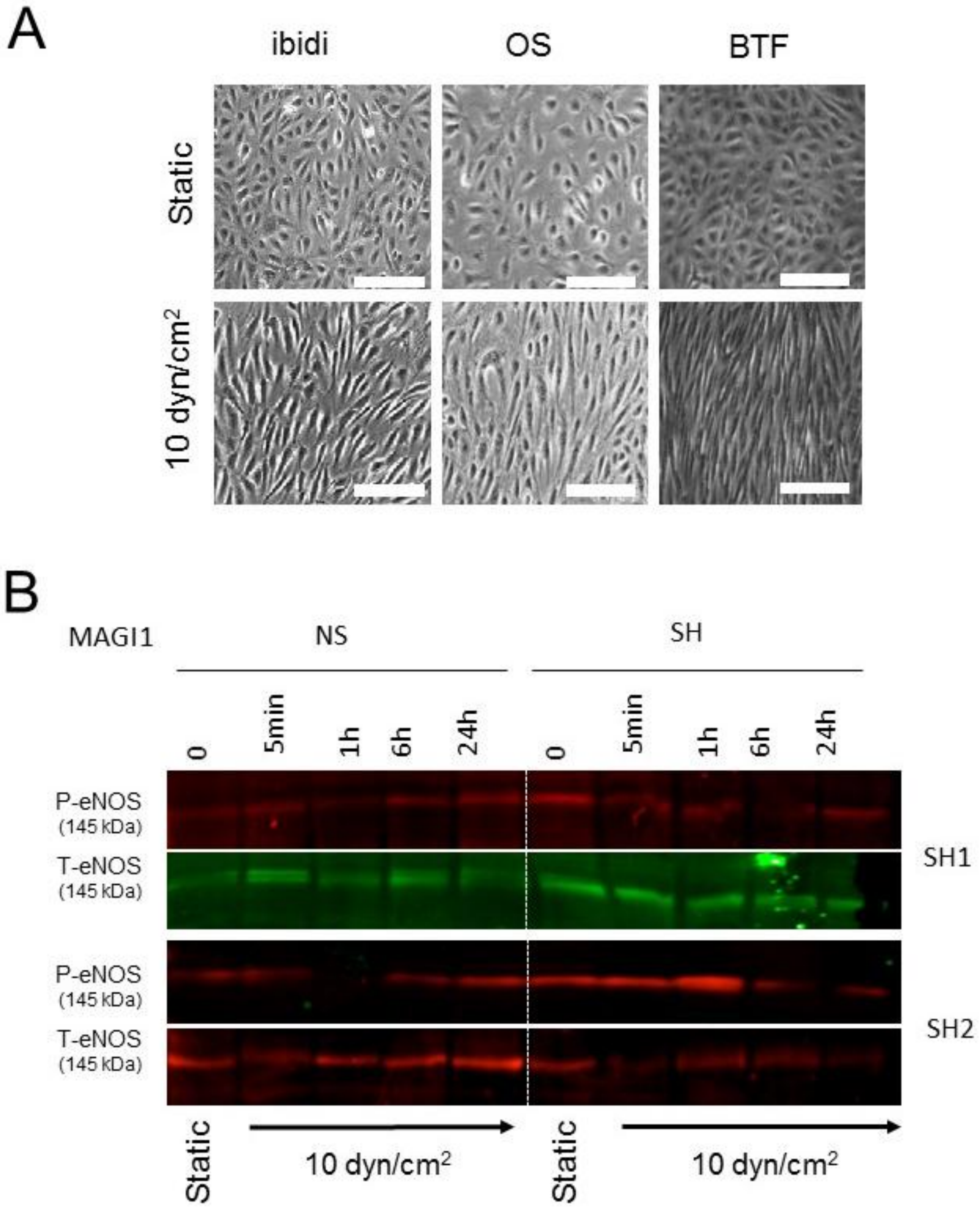

Figure S1. Flow-induced endothelial cell alignment and MAGI1- dependent eNOS phosphorylation. (A) HUVEC cultured under static conditions or exposed to 10 dyn/cm^2 fluid shear stress (flow) in the parallel plates (ibidi), orbital shaker (OS) and cone-and-plate (BTF) systems for 24 hours. HUVEC aligned to flow in the three models. Arrows: direction of flow. N= 3. Scale bar = 40 µm. (B) HUVEC non-silenced (NS) or silenced (SH2, SH4) for MAGI1 were cultured under static conditions (0) or

exposed to 10 dyn/cm^2 fluid shear stress in the orbital shaker (OS) systems for the indicated time and analysed for phosphorylated (Ser^{1177}) and total eNOS by Western blotting. MAGI silencing reduced sustained flow-induced eNOS phosphorylation.

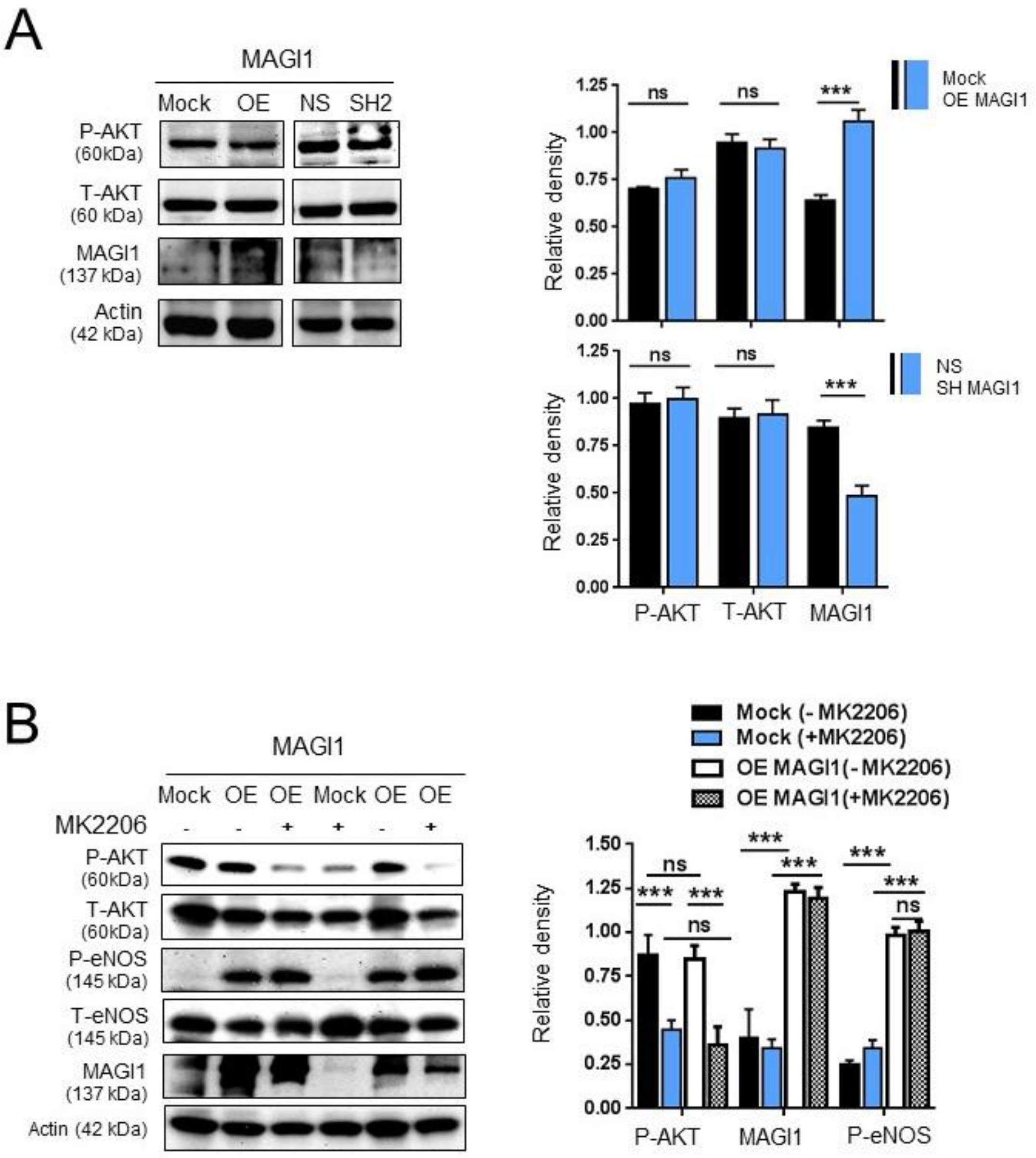

Figure S2. AKT does not contribute to MAGI1-induced eNOS phosphorylation. (A) Western blotting analysis of phosphorylated AKT (Ser^{473}) and total AKT in control HUVEC (Mock/NS) and HUVEC with silenced (SH2) or overexpressed (OE) MAGI1. N=2. (B*)* Western blotting analysis of phosphorylated or total AKT, phosphorylated

(Ser^{1177}) and total eNOS in control (Mock) and MAGI1 overexpressing (OE) HUVEC in the presence of absences of the AKT inhibitor MK2206. N=3. Beta actin is shown as loading control. Relative band quantifications by densitometry of multiple experiments are shown next to the blots. *, p<0.05; **, p<0.01; ***, p<0.001; ns, non-significant.

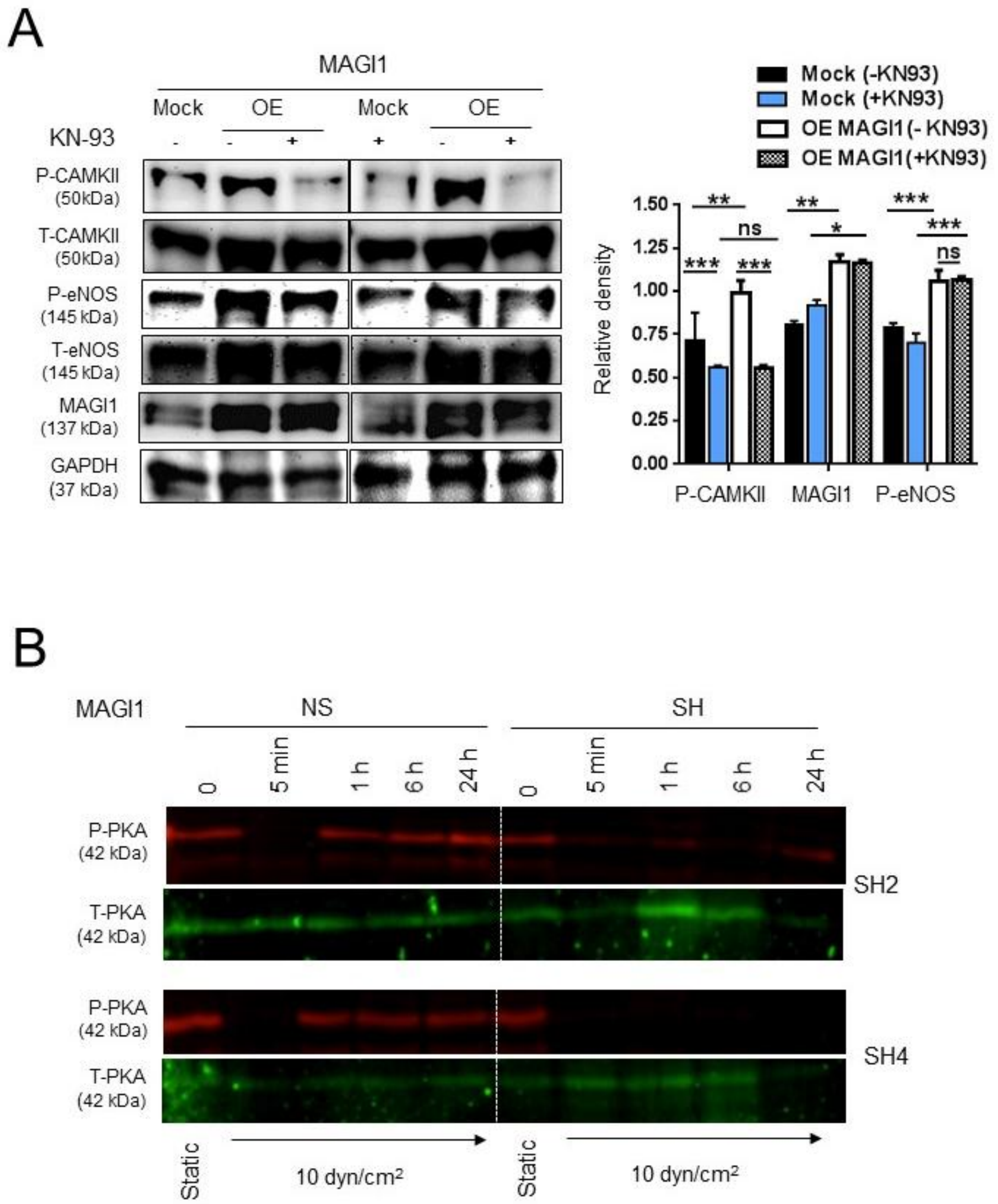

Figure S3. CAMKII does not contribute to MAGI1-induced eNOS phosphorylation. (A) Western blotting analysis of phosphorylated CAMKII (Thr^{286}), total CAMKII, phosphorylated eNOS (Ser^{1177}) and total eNOS in control (Mock) and MAGI1-overexpressing (OE) HUVEC in the presence of absence of the CAMKII inhibitor KN-93. N=3. Relative band quantifications by densitometry of multiple experiments are shown next to the blots. *, $p<0.05$; **, $p<0.01$; ***, $p<0.001$; ns, non-significant. (B) HUVEC non-silenced (NS) or silenced (SH4) for MAGI1 were cultured under static conditions (0) or exposed to 10 dyn/cm^2 fluid shear stress in the orbital shaker (OS) systems for the indicated time and analysed for phosphorylated (Thr^{197}) and total PKA levels by Western blotting. MAGI silencing reduces sustained flow-induced PKA phosphorylation.

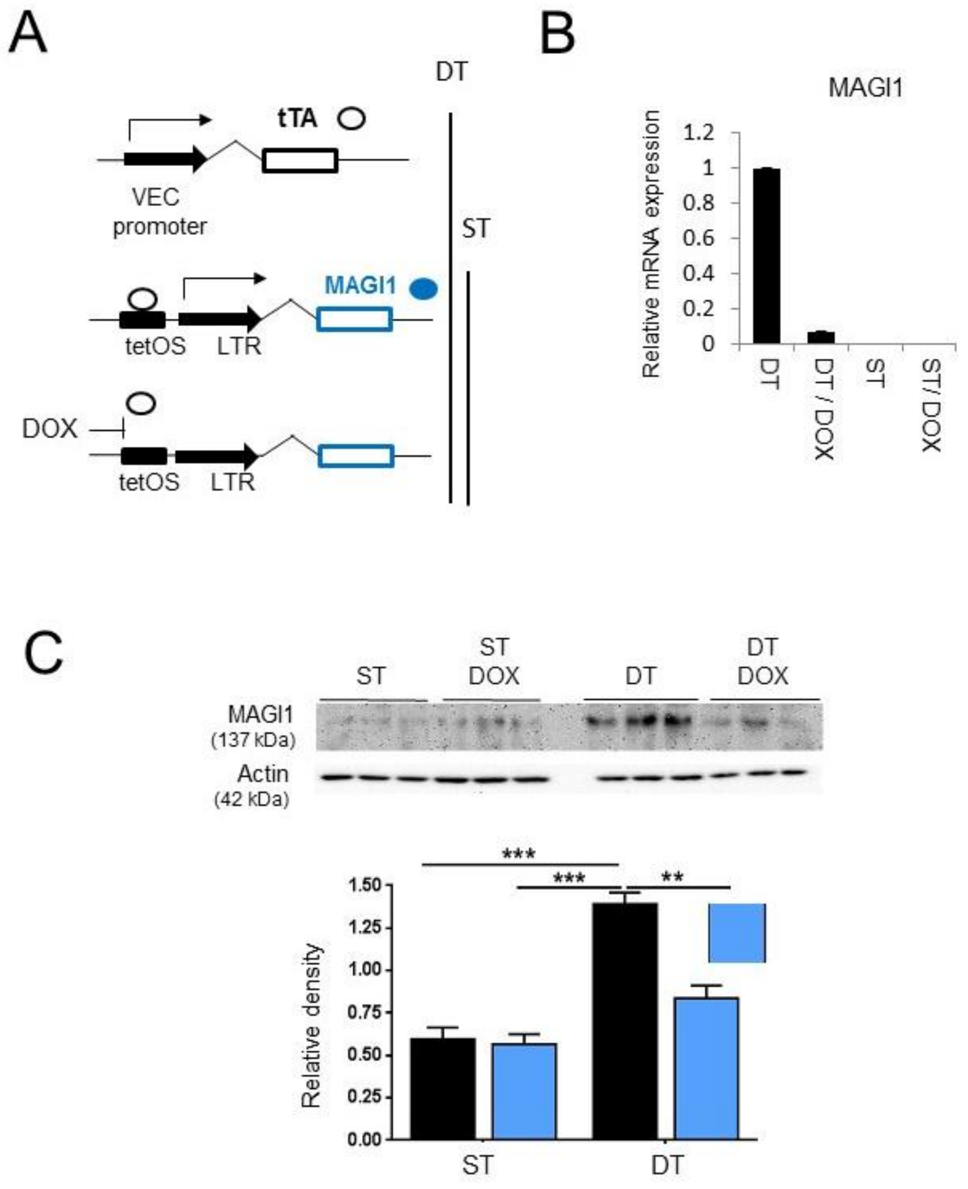

Figure S4. Transgenic expression of MAGI1 in endothelial cells. (A) Schematic representation of doxycycline (Dox) -regulated transgenic MAGI1 expression in endothelial cells. Double transgenic mice (DT) allowing doxycycline-mediated silencing of MAGI1 in mouse endothelial cells, were created by generating first a single transgenic mice (ST) line harbouring human MAGI1 cDNA under the tetOS promoter response element and crossing it with a transgenic line expressing the tet transactivator (tTA) under the control of the endothelial cell specific VE-cadherin promoter. In the absence of doxycycline DT mice constitutively expressed MAGI1 in

endothelial cells (TetOFF), addition of doxycycline blocks tTA binding to tetOS and suppressed MAGI1 expression. (B) Real time RT-PCR analysis of human MAGI1 mRNA expression in total lungs isolated from ST and DT mice, untreated or treated for 3 days with doxycycline (DOX). Bar graph gives mean values ± SD of one experiments N=3, triplicate conditions each. (C) Western blotting analysis of MAGI1 expression in lysates of total lungs isolated from ST and DT mice, untreated or treated with doxycycline (dox) for 3-days as indicated. Three mice per condition were analysed. Relative band quantification by densitometry is shown below the blots. **, $p<0.01$; ***, $p<0.001$.
